# Neuronal Organization of Deep Brain Opsin Photoreceptors in Adult Teleosts

**DOI:** 10.3389/fnana.2016.00048

**Published:** 2016-04-27

**Authors:** Chong Yee Hang, Takashi Kitahashi, Ishwar S. Parhar

**Affiliations:** Brain Research Institute, School of Medicine and Health Sciences, Monash University MalaysiaBandar Sunway, Malaysia

**Keywords:** extra-retinal, non-image-forming, evolution, neurochemical, light-sensitive

## Abstract

Biological impacts of light beyond vision, i.e., non-visual functions of light, signify the need to better understand light detection (or photoreception) systems in vertebrates. Photopigments, which comprise light-absorbing chromophores bound to a variety of G-protein coupled receptor opsins, are responsible for visual and non-visual photoreception. Non-visual opsin photopigments in the retina of mammals and extra-retinal tissues of non-mammals play an important role in non-image-forming functions of light, e.g., biological rhythms and seasonal reproduction. This review highlights the role of opsin photoreceptors in the deep brain, which could involve conserved neurochemical systems that control different time- and light-dependent physiologies in in non-mammalian vertebrates including teleost fish.

## Introduction

Light from the environment has a profound impact on almost all animals for adjusting image-forming and non-image-forming responses. Vertebrate photoreceptor cells containing photopigments, which comprise light-absorbing chromophores bound to different G-protein coupled receptor opsins, are responsible for visual and non-visual photoreception. Transmitting non-visual light signal to control physiology and behaviors (e.g., biological rhythms and seasonal reproduction) involves complex light detection systems in retinal and extra-retinal tissues (Peirson et al., 2009). The vertebrate eyes and pineal are component structures of the entire brain, which are known to be photoreceptive. However, less attention is given to the deep brain that receives light signal directly in most vertebrates. There is evidence of photic modulation of the mammalian brain activity (Vandewalle et al., 2009; Flyktman et al., 2015) and presence of deep brain opsin photoreceptors (Blackshaw and Snyder, 1999; Tarttelin et al., 2003; Kojima et al., 2011); however, direct photoreception in the mammalian brain has not been demonstrated.

Direct photoreception in the deep brain is common in non-mammalian vertebrates (Fischer et al., 2013; Kokel et al., 2013; Nakane et al., 2014), however, its relationship with physiological functions is largely unknown. Recent demonstration of the involvement of deep brain opsin photoreceptors in the control of seasonal reproduction in birds (Nakane et al., 2010, 2014) and in motor responses in larval zebrafish (Fernandes et al., 2012; Kokel et al., 2013; Friedmann et al., 2015) supports further functional studies in non-mammalian models. Ambient light conditions are known to regulate physiologies in teleost fish, e.g., skin color change (Shiraki et al., 2010), eye response (Menger et al., 2005), locomotion-related behaviors (Cahill et al., 1998; Hurd et al., 1998; Appelbaum et al., 2009; Blaser and Rosemberg, 2012), reproduction (Migaud et al., 2010), and development (Dekens et al., 2003; Dekens and Whitmore, 2008; Blanco-Vives et al., 2011; Villamizar et al., 2014). As we will discuss, multiple light-dependent physiologies in teleost fish are supposedly under the control of opsin photoreceptors in different brain regions. However, an overall distribution of different deep brain opsin photoreceptors is available only in larval zebrafish (Fernandes et al., 2013), which appears somewhat different compared with that in adults. Furthermore, although several studies have mapped opsin localization in the brain of adult teleosts, as discussed later, there are no functional studies. Here, we review the highlights of deep brain opsins identified thus far, which will lead to potential functional information of the brain photoreceptor system.

## Deep Brain Opsin Photoreceptors

Proof of concept for the deep brain photoreception in vertebrates, dated over a century ago, is attributed to an observation done by Frisch (1911). Frisch (1911) showed that European minnow lacking the eyes and pineal remain capable to change their skin color according to the change in ambient light, while a crude lesion in their diencephalon abolishes the response. Similarly, in house sparrows, an injection of India ink under their scalp abolishes their testicular growth responding to the change in photoperiod (Menaker and Keatts, 1968; Menaker et al., 1970). Furthermore, direct illumination through a fiber optic cable into the hypothalamus stimulates testicular growth in blinded ducks (Benoit and Ott, 1944). In the Japanese quail, brain tissues underneath the skull exhibit rhodopsin-like spectral sensitivity (Foster et al., 1985; Oishi and Ohashi, 1993). In non-mammalian vertebrates, with relatively light-permissive skull and skin, reaching of light signals to deep brain opsin photoreceptors is feasible (Hartwig and Veen, 1979).

Expanding genomic information and cloning efforts have discovered several groups of non-visual opsins expressed in the deep brain of vertebrates, so-called deep brain opsins. There are four subfamilies of deep brain opsins, each of which shares only 25–42% amino acid identity with existing opsin family members (Peirson et al., 2009). Most deep brain opsins present in non-mammalian vertebrate classes (i.e., birds, amphibians, reptiles, and fish) are also found in the mammalian genome: encephalopsin/panopsin (Opsin3), melanopsin (Opsin4), and neuropsin (Opsin5) (Blackshaw and Snyder, 1999; Provencio et al., 2000; Tarttelin et al., 2003; Kojima et al., 2011). Among the deep brain opsins, the earliest found is vertebrate ancient (VA)-opsin in the Atlantic salmon (Soni and Foster, 1997). VA-opsin has an elongated variant, VA-*long* (VAL)-opsin, initially reported in the zebrafish (Kojima et al., 2000); and later found as a common deep brain opsin in non-mammalian species (Halford et al., 2009). Most deep brain opsins are expressed in the retina and brain, although some [e.g., teleost-multiple-tissue (TMT)-opsin] are also expressed in peripheral tissues (Provencio et al., 1998; Moutsaki et al., 2000, 2003; Philp et al., 2000; Bellingham et al., 2002; Tarttelin et al., 2003; Kojima et al., 2011).

The presence of deep brain opsins should render brain cells direct photosensitivity. In fact, studies have shown photosensitivity of brain regions in teleosts *in vitro* and *in vivo* (Fischer et al., 2013; Kokel et al., 2013; Moore and Whitmore, 2014); also, intrinsic photosensitivity of Opsin5-positive neurons in birds *in vitro* (Nakane et al., 2014). While vertebrate visual opsins are sensitive to red, green, and blue spectrums of light (Yokoyama, 2000), deep brain opsins have distinct spectral sensitivity to blue–green spectrum and some (e.g., Opsin5) to ultraviolet light (Kojima et al., 2008; Nakane et al., 2010; Matos-Cruz et al., 2011; Sato et al., 2011; Fischer et al., 2013).

## Opsin Localization in the Brain of Teleost Fish

Among many brain regions, particularly the thalamus of adult teleosts expresses several deep brain opsin genes: *va-opsin*, *val-opsin* (*valop*), *tmt-opsin* (*tmtops*), and *opsin4* (*opn4*) isoforms. In the zebrafish thalamus, two isoforms of the *valop* genes (*valopa* and *valopb*) are actually co-expressed in single neurons constituting a major *valop* cell group located in the thalamic nuclei of the zebrafish (Hang et al., 2014). It is interesting to know that a newly defined brain region, the intercalated thalamic nucleus, contains the *valopa* and *valopb* co-expressing neurons (Hang et al., 2014). In addition, *tmtops1b*-expressing neurons might be present in the same thalamic nucleus in the zebrafish (Fischer et al., 2013). Furthermore, *opn4m1a1/m1a2*-expressing cells are co-localized with *va-opsin* expressing neurons in the dorsal thalamus of the Atlantic salmon (Sandbakken et al., 2012). The expression of multiple deep brain opsins in different fish species suggests that the thalamus is a major brain region for photoreception.

In addition to the thalamus, multiple cell groups expressing deep brain opsins are distributed in the rostral brain regions. In the telencephalon, no studies have reported the expression of deep brain opsins, whereas the granular layer of the olfactory bulb was shown to contain *tmtops1b*-expressing neurons in the medaka fish; and the preoptic area contains *tmtops2*-expressing neurons (Fischer et al., 2013). In the diencephalon, the habenula contains *va-opsin* and *opn4x1a/x1b1/x1b2*-expressing neurons, and the supraoptic/chiasmatic nucleus contains *opn4x1b1/x1b2*-expressing neurons in the Atlantic salmon and the Atlantic cod (Drivenes et al., 2003; Sandbakken et al., 2012). Furthermore, the lateral part of the lateral tuberal nucleus contains *opn4m1a1/m1a2*-expressing neurons in the Atlantic salmon (Sandbakken et al., 2012). The existence of multiple opsin photoreceptors suggests predominant photoreceptor activity and function in the rostral brain region.

The caudal brain regions including the midbrain and the hindbrain also contain deep brain opsins for photoreception in particular regions. In the midbrain, the Edinger–Westphal nucleus in the zebrafish contains a small number of *valopb*-expressing neurons (Hang et al., 2014). In the same nucleus, *tmtops1b*- and *tmtops2*-expressing neurons might co-exist (Fischer et al., 2013). The optic tectum contains *tmtops1b*-expressing neurons in the medaka fish, and the semicircular torus contains *tmtops3a*-expressing neurons (Fischer et al., 2013). In the hindbrain, the superior raphe contains *valopa*-expressing neurons in the zebrafish, and the intermediate reticular formation (IMRF) contains *valopb*-expressing neurons (Hang et al., 2014). The IMRF might also contain *tmtops1b*- and *tmtops2*-expressing neurons (Fischer et al., 2013). Expression of deep brain opsins in the cerebellum has not been reported. Generally, the number of opsin-expressing neurons in each group is small in the caudal brain of teleosts. The small groups of opsin photoreceptors might represent subfunctional involvement of photoreceptor activity in the caudal brain region.

**FIGURE 1:**
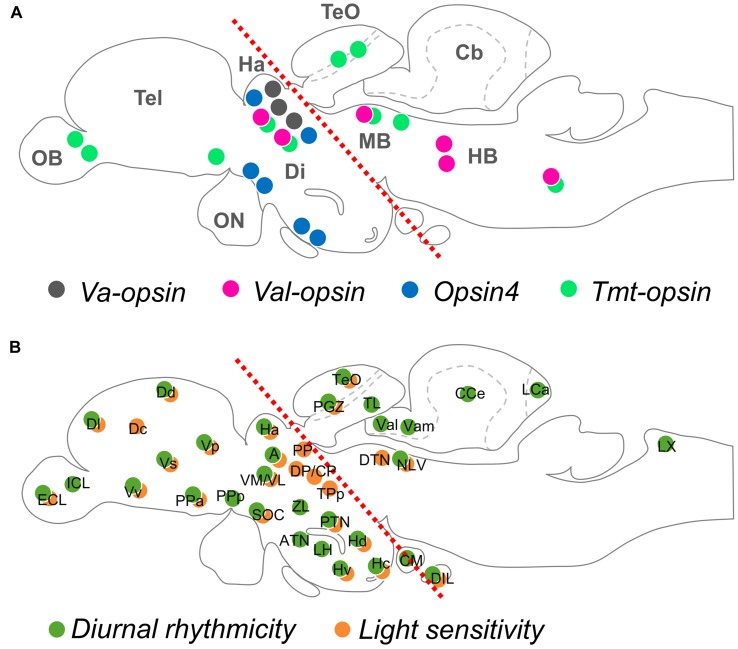
**Opsin localization and photosensitivity in the brain of adult teleost fish. (A)** An illustration mapping the neuronal groups that express deep brain photopigments, VA-opsin, VAL-opsin, Opsin4, and TMT-opsin (indicated by color dots). **(B)** An illustration mapping the diurnal rhythmicity (of clock-related gene expression; green dots) and light-sensitivity (of c-fos expression; orange dots), based on a previous work by Moore and Whitmore (2014). Red dotted lines in **(A,B)** divide the rostral and caudal regions of the adult brain, based on a previous work by Hang et al. (2015). Anatomical nomenclature used in this review are according to a brain atlas of adult zebrafish (Wullimann et al., 1996). Abbreviation (for **A**): Cb, cerebellum; Di, diencephalon; HB, hindbrain; MB, midbrain; OB, olfactory bulb; ON, optic nerve; Tel, telencephalon; TeO, optic tectum; (for **B**): A, anterior thalamic nucleus; ATN, anterior tuberal nucleus; CCe, corpus cerebellum; CM, corpus mamillare; CP, central posterior thalamic nucleus; Dc, central zone of dorsal telencephalic area; Dd, dorsal zone of dorsal telencephalic area; DIL, diffuse nucleus of the inferior lobe; Dl, lateral zone of dorsal telencephalic area; DP, dorsal posterior thalamic nucleus; DTN, dorsal tegmental nucleus; ECL, external cellular layer of olfactory bulb; Ha, habenula; Hc, caudal zone of periventricular hypothalamus; Hd, dorsal zone of periventricular hypothalamus; Hv, ventral zone of periventricular hypothalamus; ICL, inner cellular layer of olfactory bulb; LCa, caudal lobe of the cerebellum; LH, lateral hypothalamus; LX, vagal lobe; NLV, nucleus of lateral lemniscus; PGZ, periventricular gray zone; PP, periventricular pretectal nucleus; PPa, parvocellular preoptic nucleus, anterior part; PPp, parvocellular preoptic nucleus, posterior part; PTN, posterior tuberal nucleus; SOC, supraoptic/chiasmatic nucleus; TeO, optic tectum; TL, longitudinal torus; TPp, periventricular nucleus of posterior tuberculum; Val, lateral division of valvula cerebelli; Vam, medial division of valvula cerebelli; VL, ventrolateral thalamic nucleus; VM, ventromedial thalamic nucleus; Vp, posterior nucleus of ventral telencephalic area; Vs, supracommissural nucleus of ventral telencephalic area; Vv, ventral nucleus of ventral telencephalic area; ZL, zone limitans.

The current knowledge indicates that the majority of opsin-expressing neurons in adult teleosts are located in the thalamus, while there are many other minor photoreceptive brain regions (**Figure 1A**).

## Neurochemical Heterogeneity of Deep Brain Opsin Photoreceptors in Teleosts

The brain of adult teleosts has evolutionary conserved brain regions involving distinct neurotransmitter or neuropeptide systems, each of which is specialized for a particular physiological function. Neurons expressing different deep brain opsins in fish species differ in their neurochemical properties (**Table 1**). In the zebrafish, the *val-opsin* cell group in the thalamus is GABAergic; that in the Edinger–Westphal nucleus is thyrotropin-releasing hormone TRH-ergic; and that in the superior raphe is serotonergic (Hang et al., 2014). In the Atlantic salmon, the *opsin4* cell group in the habenula is serotonergic; that in the supraoptic/chiasmatic nucleus is dopaminergic; and that in the lateral part of the lateral tuberal nucleus consists of neurons expressing either corticotrophin-releasing factor or neuronal nitric oxide synthase (Sandbakken et al., 2012). In the medaka fish, the *tmt-opsin* cell group in the optic tectum and the facial motor nucleus are cholinergic (Fischer et al., 2013).

**Table 1:** Brain region-specific opsin photoreceptors in adult teleosts.

Brain region	Opsin	Fish species	Neuronal localization (*opsin isoform*)	Neuronal marker and hormone (specific region)	Reference
Telencephalon (Rostral forebrain)	TMT-opsin	Medaka *Oryzias latipes*	ICL (*tmtops1b*)	–	Fischer et al., 2013
	VA-opsin	Atlantic salmon *Salmo salar*	Ha, Th	–	Philp et al., 2000; Sandbakken et al., 2012
	VAL-opsin	Zebrafish *Danio rerio*	Th^∗^ (*valopa*/*valopb*)	GAD67	Kojima et al., 2000; Fischer et al., 2013; Hang et al., 2014
		Medaka	Th	–	Fischer et al., 2013
Diencephalon (Caudal forebrain)	Opsin4	Atlantic salmon	Ha (*opn4x1a/x1b1/x1b2*), SOC (*opn4 x1b1/x1b2*), Th and NLTl (*opn4m1a1/m1a2*)	TH (SOC), 5HT (Ha), CRF and nNOS (NLTl)	Sandbakken et al., 2012
		Atlantic cod *Gadus morhua*	Ha (*opn4x1a*), SOC (*opn4x1b*)	–	Drivenes et al., 2003
	TMT-opsin	Zebrafish	Th (*tmtops1b*)	–	Fischer et al., 2013
		Medaka	Th (*tmtops1b/2*), POA (*tmtops2*)	–	Fischer et al., 2013
Mesencephalon (Midbrain)	VAL-opsin	Zebrafish	EW^∗^ (*valopb*)	TRH	Hang et al., 2014
		Medaka	DTN	–	Fischer et al., 2013
	TMT-opsin	Zebrafish	DTN (*tmtops2b/3a*) and TeO (*tmtops1b*)	ChAT (TeO)	Fischer et al., 2013
		Medaka	DTN and TeO (*tmtops1b/2*), TSc (*tmtops3a*)	ChAT (TeO)	Fischer et al., 2013
Rhombencephalon (Hindbrain)	VAL-opsin	Zebrafish	SR (*valopa*), IMRF^∗^ (*valopb*)	pet1 (SR)	Hang et al., 2014
		Medaka	NVIIm	ChAT	Fischer et al., 2013
	TMT-opsin	Zebrafish	NVIIm (*tmtops1b/2a*)	ChAT	Fischer et al., 2013
		Medaka	NVIIm (*tmtops1b/2*)	ChAT	Fischer et al., 2013

Hyphens (-) indicate information not available. Note that VA-opsin in the zebrafish was reported to have no photosensitivity, although VA-opsin in the Atlantic salmon is blue sensitive; VAL-opsin (valop) is green sensitive; Opsin4 (opn4) and TMT-opsin (tmtops) are blue sensitive. Asterisks (*) indicate the localization of valop cells in those regions are reinterpreted by Hang et al. (2014). GAD67 is a marker for GABAergic neurons; TH, tyrosine hydroxylase or dopaminergic neurons; ChAT, choline acetyltransferase or cholinergic neurons; pet1, raphe serotonergic neurons. 5HT is 5-hydroxytryptamine or serotonin; CRF, corticotrophin-releasing factor; nNOS, nitrite oxide synthase; TRH, thyrotropin-releasing hormone. Anatomical nomenclature: DTN, dorsal tegmental nucleus; EW, Edinger–Westphal nucleus; Ha, habenula; ICL, inner cellular layer of olfactory bulb; IMRF, intermediate reticular formation; NLTl, lateral part of the lateral tuberal nucleus; NVIIm, facial motor nucleus; POA, preoptic area; SOC, supraoptic/chiasmatic nucleus; SR, superior raphe; TeO, optic tectum; Th, thalamus; TSc, central nucleus of torus semicircularis.

The neurochemical heterogeneity of deep brain opsin photoreceptors in adult teleosts supports the idea that each of them in different brain regions is involved in distinct light-dependent physiologies.

## Diurnal Rhythmicity and Light Sensitivity in the Brain of Teleost Fish

The activity of opsin photoreceptors in the brain of adult teleosts may vary according to time of day and ambient light conditions. Our recent work in the zebrafish revealed that the expression of the *valop* genes in the thalamus is rhythmic and suppressed by light, while that in the midbrain and the hindbrain is arrhythmic and not affected by light (Hang et al., 2015). This suggests region-dependent diurnal rhythmicity and light sensitivity in the brain. In fact, the expression of a rhythmic clock gene *per3*, light-responsive clock genes *cry1a* and *per2*, and a neuronal activity marker gene *c-fos* vary among various nuclei of adult zebrafish (Moore and Whitmore, 2014).

**Figure 1B** illustrates locations of diurnal rhythmicity and light sensitivity in the brain of adult zebrafish, which are mostly present in the rostral brain regions, however, less in the caudal brain regions. This is in agreement with the general localization patterns of deep brain opsin photoreceptors currently known (**Figure 1A**), and arrhythmic expression of the *valop* genes seen in the caudal brain regions in the zebrafish. Indeed, the diurnal rhythmicity and the light sensitivity of the *valop* genes in the thalamus of zebrafish shown in Hang et al. (2015) align with the diurnal expression of *per3* and light-induced *c-fos* expression in the thalamus. Besides, when observe closely, the adult zebrafish head has the upper scalp with a less pigmented anterior part (Vargas et al., 2011), which allows a wide range of the wavelength and a large amount of light reaching to the rostral brain regions underneath. Taken together, opsin photoreceptors located in the rostral and caudal brain regions may be regulated differently and serve different functionalities especially in the zebrafish physiology.

## Functional Divergence of Deep Brain Opsin Photoreceptors in Teleosts

The thalamus is a major photoreceptive brain region. Studies suggest its involvement in the control of forebrain (telencephalon) activity linking to a light-avoidance behavior in the zebrafish (Lau et al., 2011; Mueller, 2012). The telencephalon of adult teleost fish shows light sensitivity, yet no opsin expressions have been reported (**Figure 1**), and thus may receive light information from the photoreceptive thalamus. One of the major neurotransmitters GABA is associated with the thalamic *valop* cell group in the zebrafish, which is regulated by daily cycles and light (Hang et al., 2014, 2015). In contrast to that, distinct *valop* cell groups in the caudal brain are not affected by light. The evidence suggests functional divergence between the photoreceptors in the thalamus and those in other brain regions.

*Opn4*-expressing neurons in the habenula are proposed to be involved in an unidentified function of the parapineal organ; while dopaminergic *opn4*-expressing neurons in the supraoptic/chiasmatic nucleus and the lateral part of the lateral tuberal nucleus might have a role in multiple pituitary functions related to photic control of reproduction in teleost fish (Sandbakken et al., 2012). Cholinergic *tmtops*-expressing neurons reported in the optic tectum and facial motor nucleus might be photoreceptive interneurons or motorneurons (Fischer et al., 2013). TRH-(thyrotropin-releasing hormone)-ergic v*alop*-expressing neurons in the Edinger–Westphal nucleus might mediate a light-dependent eye response; while serotonergic *valop*-expressing neurons in the superior raphe could regulate anxiety-like behavior depending on ambient light conditions (Hang et al., 2014). Furthermore, in larval zebrafish, Opsin4 in the hypothalamus and unidentified opsins in the hindbrain are involved in photic motor response (Fernandes et al., 2012; Kokel et al., 2013). These photoreceptive brain regions reported in larvae might have similar functions in adult. The association of deep brain photoreceptors with distinct neurochemical systems supports their involvement in multiple physiologies in teleosts.

The diurnal and photic regulation of the *valop* genes in the thalamus demonstrated in Hang et al. (2015) is in agreement with the idea that deep brain opsin photoreceptors mediate time- and light-dependent physiology to adjust to environmental changes. It is likely that other opsins (i.e., TMT-opsin and Opsin4) co-localized in the thalamus of adult teleosts are also under the control of daily cycles and light; while opsins in other brain regions may or may not. The current neuroanatomical organization of deep brain opsins and photosensitivity in adult teleosts supports the complexity of the brain photoreceptor system to serve non-visual functions of light. However, although functional roles of deep brain opsins in larval zebrafish have been reported, no studies to date have directly shown functional roles of deep brain opsins in adult teleosts.

## Evolutionary Perspectives

So far, researchers studying the mammalian non-visual photoreception mainly examined the roles of Opsin4-expressing retinal ganglion cells (RGCs). RGCs innervate hypothalamic regions, especially the suprachiasmatic nucleus (SCN) (Hattar et al., 2002), and other brain regions including the olivary pretectal nucleus (OPN) (Hattar et al., 2006; Baver et al., 2008). The SCN is a master pacemaker (biological clock) in mammalian brain known to govern daily rhythms in physiological activities (e.g., melatonin secretion by the pineal, reproduction, and sleep). The OPN relays inputs to the Edinger–Westphal nucleus (EW), which is known to control the pupillary light reflex (Kozicz et al., 2011). In addition, the habenula (an indirect target of mammalian RGCs) exhibits light-dependent rhythmic activity (Zhao and Rusak, 2005; Hattar et al., 2006), and it is known to direct raphe serotonergic regulation of aversive mood (Hikosaka, 2010). Genetic ablation studies in mice confirmed the roles of retinal Opsin4 in circadian and pupil responses to light as well as mood (Lucas et al., 2001, 2003; Panda et al., 2002, 2003; Ruby et al., 2002; Hattar et al., 2003; LeGates et al., 2012). Note that the SCN, EW, habenula, and the raphe are also listed as photoreceptive brain regions in teleosts. Mammalian retinal Opsin4-expressing cells also innervate the thalamus, which may serve as an intermediate regulator involved in non-image-forming functions of light (Noseda et al., 2010; Hammer et al., 2015). As shown in teleosts, the thalamus is a major photoreceptive region that can mediate time- and light-dependent physiology. Taken together, while neuronal systems involved in non-visual functions of light may be evolutionarily light sensitive in vertebrates, intrinsic photosensitivity of the neuronal systems was replaced by the neuronal input from retinal photoreceptors in mammals. Therefore, studying deep brain opsin photoreceptors in teleost fish will help identify neurons responsible for non-visual light responses in mammals.

In birds, the hypothalamus is considered to be a major photoreceptive brain region, since it contains multiple deep brain opsins, i.e., VA-opsin, Opsin4, and Opsin5 (Halford et al., 2009; Kang et al., 2010; Nakane et al., 2010). These opsins in the hypothalamus have been proposed to play a role in the control of seasonal reproduction in birds (Halford et al., 2009; Kang et al., 2010; Nakane et al., 2010). The hypothesis was further supported by recent reports showing the potential Opsin5 regulation of thyroid-stimulating hormone in the pars tuberalis of the pituitary, which triggers photoperiodic response in the bird gonads (Nakane et al., 2014); and the co-expression of VA-opsin with gonadotropin-releasing hormone (GnRH), a key regulator of reproduction in the bird hypothalamus (Garcia-Fernandez et al., 2015). It is unknown whether multiple opsins co-existing in the bird hypothalamus co-operate to regulate reproduction or have distinct roles. In most vertebrates except for birds, kisspeptin neurons play a crucial role to transmit photoperiodic information to the hypothalamic GnRH-mediated reproductive system (Parhar et al., 2012). It will be interesting to examine whether GnRH neurons or the kisspeptin neurons are directly photoreceptive in teleost fish, or they are innervated by other photoreceptive nuclei (e.g., the thalamus).

The major brain regions and the neurochemical systems associated with non-visual functions of light are fundamentally conserved among vertebrates. Light-dependent alteration in brain neurotransmitter systems (e.g., hypothalamic dopaminergic and raphe serotonergic) involved in light-dependent physiologies (e.g., adjusting skin pigmentation and depression-like behaviors) has been reported in *Xenopus* and rats (Dulcis and Spitzer, 2008; Gonzalez and Aston-Jones, 2008; Dulcis et al., 2013). However, the involvement of non-visual (deep brain) opsin photoreceptors in that remains largely unknown. In *Xenopus*, only Opsin4 photoreceptor was localized especially in the supraoptic/chiasmatic nucleus (Provencio et al., 1998). While rod/cone-like-opsin and (pineal) pin-opsin immunoreactivity in the hypothalamus was subsequently reported in amphibians and reptiles, information about known deep brain opsin photoreceptors localized in their brain is limited (Yoshikawa et al., 1998; Okano et al., 2000; Vigh et al., 2002; Pasqualetti et al., 2003). In mice, a study mapped the distribution of Opsin3 photoreceptors in the cortex, hypothalamus, thalamus, cerebellum, and the spinal cord (Blackshaw and Snyder, 1999). How light can reach directly to deep brain photoreceptors through the mammalian skull remains questionable, but there might be an alternative pathway. In fact, through the ear canal, promising effects of transcranial light on plasma monoamine levels and Opsin3 expression in the brain of adult mice was recently observed (Flyktman et al., 2015). Note that some Opsin3-expressing brain regions (i.e., hypothalamus and thalamus) are photoreceptive brain regions in teleosts. Besides, a recent study identified novel vertebrate opsins and showed their expression in brain regions including the cerebellum of adult zebrafish (Davies et al., 2015). Further studies in teleosts will help elucidate conserved mechanisms that non-visual (deep brain) opsin photoreceptors mediate time- and light-dependent physiologies involving neurotransmitter systems.

## Concluding Remarks

Deep brain opsin photoreceptors in multiple brain regions involving conserved neurochemical systems can mediate the control of time- and light-dependent physiologies in adult teleost fish. Knowing the detailed neuroanatomical organization of individual deep brain opsins in vertebrates (especially non-mammals) would help advance our understanding of the physiological importance of non-visual photoreception.

## Author Contributions

CYH, TK, and ISP conceived the study. CYH prepared the figure and table, and wrote the manuscript. TK and ISP gave constructive comments of this work. All authors reviewed the manuscript.

## Conflict of Interest Statement

The authors declare that the research was conducted in the absence of any commercial or financial relationships that could be construed as a potential conflict of interest.
